# Priming of conflicting motivational orientations in heavy drinkers: robust effects on self-report but not implicit measures

**DOI:** 10.3389/fpsyg.2015.01465

**Published:** 2015-10-02

**Authors:** Lisa C. G. Di Lemma, Joanne M. Dickson, Pawel Jedras, Anne Roefs, Matt Field

**Affiliations:** ^1^Department of Psychological Sciences, University of LiverpoolLiverpool, UK; ^2^UK Centre for Tobacco and Alcohol StudiesLiverpool, UK; ^3^Clinical Psychological Science, Maastricht UniversityMaastricht, Netherlands

**Keywords:** alcohol, ambivalence, approach, automatic, avoidance, implicit, thought suppression

## Abstract

We report results from three experimental studies that investigated the independence of approach and avoidance motivational orientations for alcohol, both of which operate within controlled and automatic cognitive processes. In order to prime their approach or avoidance motivational orientations, participants watched brief videos, the content of which (positive or negative depictions of alcohol, or neutral) varied by experimental group. Immediately after watching the videos, participants completed self-report (Approach and Avoidance of Alcohol Questionnaire; all studies) and implicit (visual probe task in study 1, stimulus-response compatibility task in studies 2 and 3) measures of alcohol-related approach and avoidance. In study 3, we incorporated an additional experimental manipulation of thought suppression in an attempt to maximize the influence of the videos on implicit measures. Findings were consistent across all three studies: increases in self-reported approach inclinations were mirrored by decreases in avoidance inclinations, and vice versa. However, a combined analysis of data from all studies demonstrated that changes in approach inclinations were partially independent of changes in avoidance inclinations. There were no effects on implicit alcohol-related processing biases, although methodological issues may partially account for these findings. Our findings demonstrate that subjective approach and avoidance inclinations for alcohol tend to fluctuate in parallel, but changes in approach inclinations may be partially independent from changes in avoidance inclinations. We discuss methodological issues that may partially account for our findings.

## Introduction

According to the ambivalence model of craving (Breiner et al., 1999; McEvoy et al., 2004), the decision to consume alcohol is determined by the balance between motivational inclinations to indulge (“approach”) and to abstain (“avoidance”). Approach and avoidance inclinations might arise from the desire for intoxication or the wish to keep a clear head for the next day, respectively. Motivational conflict (or ambivalence), which plays an important role in alcohol use disorders and their treatment (Hettema et al., 2005), arises when a person has the motivation to drink and to abstain at the same time. Importantly, these motivational orientations can operate in both controlled (or explicit) and automatic (or implicit) cognitive processes. Controlled processes are rule-based and reflective, they operate within conscious awareness and they can be assessed with self-report measures. Automatic processes are activated spontaneously and they are typically assessed with indirect tasks such as computerized measures of attentional bias and automatic approach tendencies (Stacy and Wiers, 2010). One theoretical model proposed that subjective craving (a controlled process) and attentional bias (an automatic process) have reciprocal causal influences on each other (Field and Cox, 2008), although an alternative account is that automatic and controlled processes are both outputs of underlying processes that cannot be measured directly (motivational orientations; see Christiansen et al., 2015). In this paper we report results from three studies in which we experimentally manipulated motivational orientations for alcohol in order to thoroughly investigate the independence of approach and avoidance in both controlled and automatic processing.

Regarding controlled processes, the Approach and Avoidance of Alcohol Questionnaire (AAAQ; McEvoy et al., 2004) was developed to capture the strength of approach and avoidance inclinations for alcohol. The initial factor analysis of non-dependent drinkers' responses on the AAAQ yielded three subscales, two representing approach inclinations (inclined-indulgent and obsessed-compelled subscales, corresponding to mild and strong inclinations, respectively), and one representing inclinations to avoid drinking alcohol (the resolved-regulated subscale). Subsequent studies employed the AAAQ with different populations of drinkers and performed factor analysis on participants' responses. Each of these studies confirmed that approach and avoidance represent distinct underlying factors, although some studies with alcohol dependent patients (Klein et al., 2007; Schlauch et al., 2013c; but see Klein and Anker, 2013) identified a single underlying factor to approach inclinations rather than the qualitative distinction between mild and strong approach that was reported in the initial study (McEvoy et al., 2004). Many of these studies demonstrated that both approach and avoidance inclinations are independently associated with drinking-related variables. For example, approach and avoidance inclinations account for unique variance in quantity and frequency indices of alcohol consumption in both non-dependent (McEvoy et al., 2004) and alcohol dependent (Klein et al., 2007) drinkers. Approach and avoidance inclinations also have differential predictive validity in alcohol-dependent patients: following treatment, relapse to drinking is predicted by the strength of approach inclinations, but avoidance inclinations are not predictive (Schlauch et al., 2012; Klein and Anker, 2013; see also Schlauch et al., 2013c). On the other hand, avoidance inclinations (but not approach inclinations) predict the likelihood of entering into and engaging with treatment (Schlauch et al., 2012). Taken together, these findings provide support for the ambivalence model of craving (Breiner et al., 1999) because they demonstrate that self-reported approach and avoidance inclinations for alcohol are separable constructs that are uniquely associated with past and future drinking behavior (see also Curtin et al., 2005; Schlauch et al., 2013a,b).

Regarding automatic processes, there is evidence for co-existence of appetitive (approach) and aversive (avoidance) alcohol-related processing biases in problem drinkers in a variety of sub-domains, including affective associations (Dickson et al., 2013), attentional bias (Stormark et al., 1997), and approach and avoidance tendencies (Barkby et al., 2012). Regarding attentional bias, heavy drinkers who are not seeking treatment have an attentional bias for alcohol cues (Townshend and Duka, 2001; Field et al., 2004). The strength of this attentional bias is reliably associated with the strength of subjective craving (Field et al., 2009) and is potentiated by experimental manipulations that increase the motivation to drink, such as induction of negative mood and exposure to alcohol-related cues (see Field and Cox, 2008). By contrast, alcohol-dependent patients who are tested in treatment contexts show initial attentional bias that is quickly followed by attentional avoidance (Stormark et al., 1997; Noël et al., 2006; Townshend and Duka, 2007; Vollstädt-Klein et al., 2009; Field et al., 2013). The latter pattern of attentional bias may reflect ambivalence, with appetitive motivational processes mapped to the initial attentional bias and aversive motivational processes mapped on to the subsequent attentional avoidance (see Field et al., 2013, for discussion). Consistent with this interpretation, a recent eye tracking study demonstrated that heavy drinkers who were identified as ambivalent (as assessed with the AAAQ) had an approach-avoidance pattern of attentional bias for alcohol cues (i.e., the initial attentional bias quickly followed by attentional avoidance that is characteristic of alcohol-dependent patients), whereas heavy drinkers who were not ambivalent maintained their attentional bias for alcohol cues (Lee et al., 2014).

Automatic approach and avoidance tendencies evoked by alcohol-related cues have been assessed with the alcohol-related stimulus-response compatibility (SRC) task (Field et al., 2008) and related tasks (Wiers et al., 2009). These tasks reveal that in heavy drinkers who are not seeking treatment, alcohol cues evoke automatic approach tendencies (Field et al., 2008, 2011; Wiers et al., 2009; Christiansen et al., 2012; Sharbanee et al., 2013a,b; Kersbergen et al., 2015), and in some studies the strength of these approach tendencies was associated with the strength of subjective craving (Field et al., 2005, 2008). A different pattern is seen in alcohol-dependent patients: one study reported no reliable tendency to approach or avoidance (Barkby et al., 2012) whereas another study found an automatic avoidance tendency, the strength of which was predictive of subsequent relapse (Spruyt et al., 2013). One explanation for these findings is that the standard version of the SRC task yields an index of automatic approach that is *relative* to avoidance. This means that the pattern that is observed in heavy drinkers who are not seeking treatment (Field et al., 2008, 2011; Christiansen et al., 2012; Kersbergen et al., 2015) could be attributed to strong automatic approach, weak automatic avoidance, or a combination of the two. Among alcohol-dependent patients, if alcohol cues simultaneously evoke strong automatic approach at the same time as strong automatic avoidance, this may explain why this population display either no overall bias (Barkby et al., 2012) or an avoidance bias (Spruyt et al., 2013) depending on the strength of their motivational orientations to avoid alcohol at the time of testing.

Findings from the cross-sectional and prospective studies described above are consistent with the ambivalence model (Breiner et al., 1999) because they suggest that approach and avoidance motivational orientations for alcohol may exist independently of each other, rather than lying at opposite ends of a single continuum. More compelling evidence for the independence of approach and avoidance can be derived from experimental studies that attempt to influence one motivational orientation (approach or avoidance) in order to investigate if the opposing motivational orientation is (un)affected. Regarding automatic processes, we recently demonstrated that subliminal priming of approach or avoidance motivational orientations for alcohol had no effect on attentional biases or automatic approach or avoidance tendencies, although methodological issues complicated interpretation of those findings (Baker et al., 2014). Regarding controlled processes, several studies investigated the effects of exposure to alcohol cues on self-reported approach and avoidance inclinations for alcohol, and all reported findings that were suggestive of partially independent approach and avoidance responses to those cues (Curtin et al., 2005; Jones et al., 2013; Schlauch et al., 2013a,b). For example, in one study exposure to alcohol cues (pouring, holding, and sniffing a beer) led to increases in approach inclinations (AAAQ inclined-indulgent and obsessed-compelled subscales), but avoidance inclinations (AAAQ resolved-regulated subscale) were unaffected (Jones et al., 2013).

Although these studies are informative, a more rigorous experimental test of the independence of approach and avoidance would be to contrast the effects of experimental manipulations that are intended to increase approach or avoidance motivational orientations for alcohol. To achieve this, we were inspired by methods used in a previous study (Roefs et al., 2006) in which participants' automatic processing of food-related words was assessed in contexts that were intended to activate either approach (focusing on the preparation of a tasty meal) or avoidance (focusing on the importance of a healthy diet, and therefore avoiding unhealthy foods). In the present studies, participants viewed short videos that depicted either the positive or negative aspects of alcohol consumption, which should in principle activate approach or avoidance, respectively. Control groups of participants viewed videos that were unrelated to alcohol consumption. Immediately after watching the videos, participants completed the AAAQ (all studies) followed by computerized measures of attentional bias (study 1) and automatic approach and avoidance tendencies (studies 2 and 3). In addition, in study 3 we investigated if thought suppression (see Moss et al., 2015) would moderate the influence of videos on implicit measures.

Our general hypotheses were that the video depicting the positive consequences of alcohol consumption would increase self-reported approach (inclined-indulgent and obsessed-compelled subscales of the AAAQ) and indices of automatic approach (attentional bias in study 1, automatic approach tendencies in studies 2 and 3), but would not influence self-reported and automatic avoidance, as assessed by the resolved-regulated subscale of the AAAQ and attentional avoidance (study 1) and automatic avoidance tendencies (studies 2 and 3), respectively. By contrast, the video depicting the negative consequences of alcohol consumption would increase self-reported and automatic indices of avoidance, but indices of approach would be unaffected.

## Study 1

The alcohol-related visual probe task (see Field et al., 2004) is a computerized measure of attentional bias that can distinguish between attentional bias toward and attentional bias away from alcohol-related pictorial stimuli (hereafter referred to as *attentional avoidance*). In each trial of the task, an alcohol-related picture and a matched neutral picture are briefly presented on opposite sides of a computer screen before a visual probe replaces one of the pictures. Participants' manual reaction times to probes are used to infer biases in the allocation of visuospatial attention. An attentional bias for alcohol cues is inferred if the participant is faster to react to probes that replace alcohol pictures (congruent trials), rather than probes that replace neutral pictures (incongruent trials). If, however, this pattern is reversed (i.e., if the participant is faster to respond on incongruent trials), this is interpreted as attentional avoidance of alcohol cues. Biases in automatic attentional capture or delayed disengagement of attention can be inferred by comparing reaction times on these trials with those on other trials in which only neutral pictures are presented (Koster et al., 2004; see Baker et al., 2014). Although the literature on group differences is inconsistent (see Field and Cox, 2008), several studies demonstrated that heavy drinkers who are not seeking treatment have an attentional bias for alcohol cues when those cues are presented for 500 ms or longer (Townshend and Duka, 2001; Field et al., 2004; Baker et al., 2014), and this has been corroborated by studies of eye movements toward those cues (Lee et al., 2014). Conversely, alcohol-dependent patients who are tested in treatment settings show initial attentional bias for briefly-presented alcohol cues (50–100 ms), that is followed by attentional avoidance when those cues are presented for longer periods (upwards of 500 ms; Stormark et al., 1997; Noël et al., 2006; Townshend and Duka, 2007; Vollstädt-Klein et al., 2009; Field et al., 2013).

In the present study, participants watched a brief video that depicted either the positive consequences of alcohol consumption (alcohol-positive group), the negative consequences of alcohol consumption (alcohol-negative group), or that had no alcohol-related content (control group). Immediately after watching the video, participants completed the AAAQ and an alcohol-related visual probe task in which picture pairs were presented for 50 or 500 ms. We hypothesized that, relative to the control group, participants in the alcohol-positive group would have elevated scores on the inclined-indulgent and obsessed-compelled subscales of the AAAQ, and elevated attentional bias for alcohol cues presented for both 50 and 500 ms; however, scores on the resolved-regulated subscale of the AAAQ would not differ between alcohol-positive and control groups. By contrast, compared to the control group, participants in the alcohol-negative group would have elevated scores on the resolved-regulated subscale of the AAAQ and would exhibit an “approach-avoidance” pattern of attentional bias on the visual probe task, with bias toward alcohol cues presented for 50 ms followed by attentional avoidance of those cues presented for 500 ms; however, scores on the inclined-indulgent and obsessed-compelled subscales of the AAAQ would not differ between the alcohol-negative and control groups.

### Methods

#### Participants

Ninety participants (69 Female, mean age 21.70, *SD* = 5.04) were recruited from the students and staff at the University of Liverpool via online and poster advertising. Inclusion criteria included fluency in English, age between 18 and 45, normal or corrected-to-normal vision and self-reported alcohol consumption in excess of the current UK government guidelines for safe drinking (these are 14 units per week for females and 21 units per week for males, where 1 unit equals 8 g of alcohol). Exclusion criteria included any history of alcohol use disorders. Participants who had taken part in studies 2 or 3 were ineligible to participate. All participants provided informed consent before taking part in the study, which was approved by the University of Liverpool Research Ethics Committee.

#### Materials

##### Self-report measures

##### Timeline followback drinking diary (Sobell and Sobell, 1992)

Participants indicated their alcohol consumption over the previous 2 weeks. From this, we were able to calculate the total amount of alcohol consumed in standard UK units.

##### Alcohol use disorders identification test (AUDIT; Saunders et al., 1993)

This 10-item self-report questionnaire contains questions about frequency and quantity of alcohol consumption, and alcohol-related problems and harms. It yields a total score ranging between 0 and 40, with scores of 8 or above indicative of hazardous drinking.

##### Approach and avoidance of alcohol questionnaire, right now version (AAAQ; McEvoy et al., 2004)

This 14-item questionnaire assesses subjective tendencies to approach or avoid drinking at that moment in time. Respondents are asked to rate how strongly they agree with each item on a 9-point Likert scale, from 0 (not at all) to 8 (very strong). There are three underlying sub-scales: “Inclined-Indulgent” (mild approach, akin to desire to drink) “Obsessed-Compelled” (strong approach, akin to obsessive thoughts about drinking); and “Resolved-Regulated” (motivation to avoid drinking).

##### Positive and negative affect schedule (PANAS; Watson et al., 1988)

The PANAS is a 20-item Likert scale that yields scores on positive affect (PA) and negative affect (NA). Results are reported in the Supplementary Materials.

##### Video questionnaire

This eight-item questionnaire was developed to measure participants' perception of and engagement with the videos. Participants responded to each item on a 5-point Likert scale, with labels ranging from “strongly disagree” to “strongly agree.” Items are shown in Tables S2A–C.

##### Visual probe task (for similar tasks see Field et al., 2004; Koster et al., 2004)

This task was programmed in Psychopy v.1.74 (Peirce, 2007) and was administered on a desktop computer with a 15-inch monitor. On each trial, a small white fixation cross was presented in the center of the screen for 500 ms. Immediately after offset, a pair of pictures (each 65 mm high × 80 mm wide) was presented on the left and right of the screen, 130 mm apart, for either 50 or 500 ms. Immediately after the screen was cleared, the visual probe (a small white arrow that pointed up or down) was presented on either the left or right side of the screen, in the position that had been occupied by one of the pictures. The probe remained on the screen until participants made a response by pressing a key labeled “up” or “down” on the computer keyboard.

Participants were instructed to rest the index fingers of their left and right hands on the “up” and “down” keys, to fixate on the fixation cross at the beginning of each trial, and to rapidly categorize the visual probe as soon as it appeared. The latency and accuracy of responses were recorded. There was an initial practice block of 10 trials in which four pairs of affectively neutral pictures, taken from the International Affective Picture System (IAPS; Lang et al., 2008) were presented. The main block of trials then followed, and this comprised two different types of trials: alcohol-neutral trials, and neutral-neutral trials. For alcohol-neutral trials, a set of seven alcohol-related pictures were each paired with neutral pictures that depicted items of stationery. We used a subset of picture pairs that had been used in earlier studies (Field et al., 2011; Barkby et al., 2012) and the pictures in each pair were matched on perceptual characteristics including brightness and complexity. On neutral-neutral trials, we used four pairs of affectively neutral pictures from the IAPS, as described above. During the main block of trials, there were 112 alcohol-neutral trials and 64 neutral-neutral trials. Each picture pair was presented 16 times, and picture location (left or right), stimulus onset asynchrony (SOA; 50 or 500 ms), probe position (left or right) and probe type (up or down arrow) were counterbalanced for all picture pairs. Trials were presented in a random order.

##### Video stimuli

We created three different videos in order to manipulate participants' inclinations to drink alcohol or to refrain from drinking. Videos were created in Windows Movie Maker (version 2.6) and were presented in Windows Media Player (version 7) player in full-screen mode on the computer. Participants wore headphones while watching the videos, all of which were 3 min and 45 s in duration. All video files are available from the Corresponding Author on request.

The *alcohol-positive* video was intended to evoke motivational inclinations to approach alcohol. It comprised still images depicting people having fun while drinking alcohol, together with some text slides that provided information about the positive consequences of drinking and was accompanied by an upbeat soundtrack. The *alcohol-negative* video was intended to evoke motivational inclinations to avoid alcohol. It comprised still images depicting the negative consequences of drinking, including scenes of alcohol-related violence and vomiting, and other slides depicting graphic government advertisements that warned of the consequences of drink-driving and alcohol-related organ damage and was accompanied by a downbeat soundtrack. The *neutral* video comprised still photos of office equipment and furniture, and was accompanied by non-descript jazz music. All images were obtained using a Google Images search.

#### Procedure

Participants were randomly allocated to experimental condition. They were tested in a laboratory in the Department of Psychological Sciences at the University of Liverpool. After providing informed consent participants completed the timeline follow back drinking diary, AUDIT, AAAQ, and PANAS (time 1). Then, participants put on the headphones and watched one of the videos (depending on experimental condition), before completing the Video Questionnaire and the AAAQ and PANAS again (time 2). Finally, participants completed the visual probe task. After completing the study, participants were debriefed and offered either course credit or a £5 Shopping Voucher to compensate them for their time.

### Results

#### Group characteristics

Participants reported consuming 20.55 (*SD* = 11.53) units of alcohol per week, and the mean score on the AUDIT was 12.18 (*SD* = 5.28). There were no between-group differences in weekly alcohol consumption or AUDIT scores (Kruskal–Wallis tests *ps* > 0.09), although there was a trend for participants in the alcohol-positive group to be older than participants in the other two groups (Kruskal–Wallis *p* = 0.05). There were no group differences in gender ratio (χ^2^ = 0.45, *p* > 0.1).

#### Effects of video manipulation on AAAQ ratings (Figure 1A)

AAAQ ratings were analyzed using a mixed design ANOVA, with within-subject factors of sub-scale (3: inclined-indulgent, obsessed-compelled, resolved-regulated), time (2: before video, after video), and group (3: alcohol-positive, alcohol-negative, control). The sub-scale x time x group interaction was statistically significant [*F*_(4, 174)_ = 27.05, *p* < 0.001]. Subsequent *post-hoc* ANOVAs confirmed that the time x group interaction was significant for all three sub-scales [inclined-indulgent *F*_(2, 87)_ = 25.29, *p* < 0.001]; obsessed-compelled *F*_(2, 87)_ = 5.72, *p* < 0.01; resolved-regulated *F*_(2, 87)_ = 28.32, *p* < 0.001].

**Figure 1:**
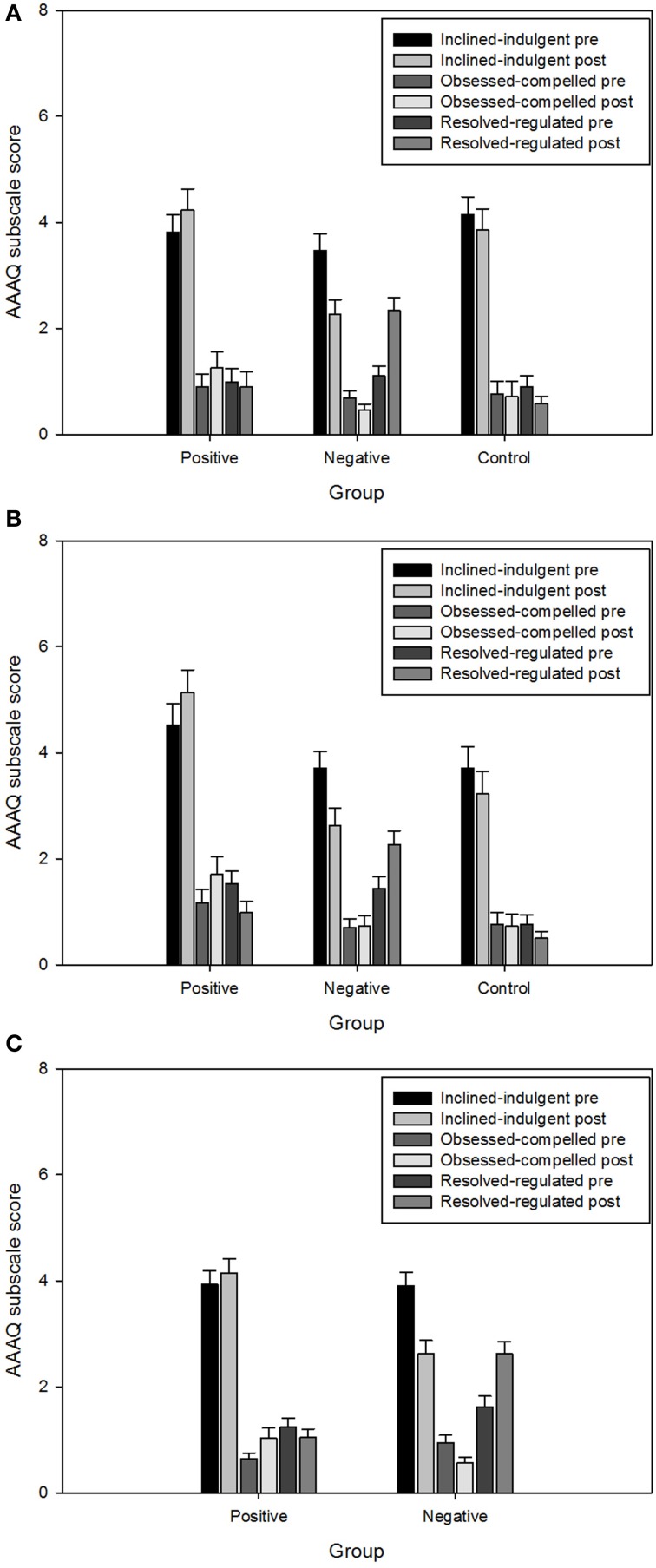
**Responses on the AAAQ in study 1 (A), study 2 (B), and study 3 (C)**. Responses range from 0 to 8. Values are means (± SEM).

There were no group differences on any of the AAAQ sub-scales before participants watched the video [inclined-indulgent *F*_(2, 89)_ = 1.12, *p* > 0.1; obsessed-compelled *F*_(2, 89)_ = 0.26, *p* > 0.1; resolved-regulated *F*_(2, 89)_ = 0.22, *p* > 0.1]. As predicted, groups differed on all three sub-scales after watching the video, [inclined-indulgent *F*_(2, 89)_ = 9.13, *p* < 0.001; resolved-regulated *F*_(2, 89)_ = 17.57, *p* < 0.01], although this fell short of significance for the obsessed-compelled sub-scale [*F*_(2, 89)_ = 2.93, *p* = 0.059]. *Post-hoc* LSD contrasts confirmed that scores on both the inclined-indulgent and obsessed-compelled sub-scales were higher in the alcohol-positive group compared to the alcohol-negative group (*ps* < 0.01), but this pattern was reversed for the resolved-regulated subscale (*p* < 0.01). The direct test of our hypotheses requires contrasts between these groups and the control group. These contrasts revealed that alcohol-positive and control groups did not differ on any subscale (*p* > 0.1). However, scores on the resolved-regulated subscale were higher, and scores on the inclined-indulgent subscale lower, in the alcohol-negative compared to the control group (*ps* < 0.01). Alcohol-negative and control groups did not differ on the obsessed-compelled subscale (*p* > 0.1).

Paired-samples *t*-tests revealed that among participants in the alcohol-positive group, scores on the inclined-indulgent and obsessed-compelled sub-scales increased after watching the video [*t*_(28)_ = 2.92, *p* < 0.01 and *t*_(28)_ = 2.29, *p* < 0.05], whereas scores on the resolved-regulated sub-scale did not change [*t*_(28)_ = 0.61, *p* > 0.1]. A different pattern was seen in the alcohol-negative group: inclined-indulgent and obsessed-compelled scores decreased [*t*_(31)_ = 6.41, *p* < 0.001 and *t*_(31)_ = 2.24, *p* < 0.05], whereas scores on the resolved-regulated sub-scale increased [*t*_(31)_ = 6.34, *p* < 0.001]. In the control group, scores on both the inclined-indulgent and resolved-regulated sub-scales decreased after watching the video, although the former failed to reach significance [*t*_(28)_ = 1.95, *p* = 0.06 and *t*_(28)_ = 2.66, *p* < 0.05]; scores on the obsessed-compelled sub-scale did not change [*t*_(29)_ = 0.30, *p* > 0.1].

We also re-ran the omnibus Three-Way ANOVA on AAAQ scores but added PANAS positive and PANAS negative affect after the video as covariates. The three way interaction sub-scale × time × group remained statistically significant [*F*_(4, 170)_ = 17.25, *p* < 0.001]. Therefore, statistically controlling for positive and negative mood at the time did not modify the influence of the videos on the AAAQ.

#### Visual probe task (Table 1)

Data were analyzed in accordance with previous studies (e.g., Field et al., 2004). Firstly, trials with errors were discarded, and then outlying reaction times were removed if they were faster than 200 ms, slower than 2000 ms, and then if they were more than three standard deviations above the individual mean. All data from three participants were excluded as they had an outlying high rate (>28%) of missing data due to errors and outliers. For the remainder of the sample, on average 7% of trials were missing due to errors and a further 1% due to outliers, and these values did not differ between groups (*ps* > 0.1).

**Table 1:** **Mean reaction times (in milliseconds) from the different trials of the visual probe task in study 1**.

	**Alcohol-positive**	**Alcohol-negative**	**Control**
**50 ms TRIALS**			
Congruent alcohol	732.20 ± 59.29	725.82 ± 70.42	710.73 ± 67.41
Incongruent alcohol	734.37 ± 58.31	726.36 ± 73.72	699.75 ± 67.06
Neutral-neutral	729.02 ± 53.73	729.39 ± 73.26	699.35 ± 65.39
**500 ms TRIALS**			
Congruent alcohol	669.01 ± 59.49	670.54 ± 62.12	654.20 ± 68.86
Incongruent alcohol	680.24 ± 43.37	670.88 ± 62.30	664.84 ± 60.43
Neutral-neutral	675.89 ± 48.14	671.86 ± 67.49	648.36 ± 73.90

*Values are means ± SD*.

Mean reaction times for different trial types and SOAs were analyzed using a 3 × 2 × 3 mixed design ANOVA, with within-subject factors of trial type (3: congruent alcohol trials, incongruent alcohol trials, neutral-neutral trials) and SOA (2: 50, 500 ms), and a between-subjects factor of group. The predicted trial type x SOA x group interaction was not statistically significant [*F*_(4, 170)_ = 1.16, *p* > 0.1]. There was a significant main effect of SOA [*F*_(1, 85)_ = 390.10, *p* < 0.001], indicating faster reaction times on 500 ms trials compared to 50 ms trials. Importantly, the non-significant main effect of trial type [*F*_(2, 84)_ = 1.09, *p* > 0.1], and trial type × SOA interaction [*F*_(2, 84)_ = 2.12, *p* > 0.1] demonstrate that there was no reliable attentional bias for alcohol cues overall, at either SOA.

### Discussion

Overall, results from this study did not support the independence of approach and avoidance orientations for alcohol in either controlled or automatic processes. Data from the AAAQ could be interpreted as independence of self-reported approach and avoidance inclinations for alcohol after watching a video depicting the positive consequences of alcohol consumption, because participants who watched this video reported an increase in self-reported approach inclinations (inclined-indulgent and obsessed-compelled subscales) but no corresponding reduction in avoidance inclinations (the resolved-regulated subscale). However, a video that depicted the negative consequences of alcohol consumption prompted an increase in self-reported avoidance inclinations (the resolved-regulated subscale) in parallel with a decrease in self-reported approach inclinations (the inclined-obsessed and resolved-regulated subscales). Comparisons between these groups and a control group revealed that approach and avoidance inclinations were similar in the control group and the group that had watched the video depicting the positive consequences of alcohol consumption, whereas approach inclinations were lower, and avoidance inclinations higher, in the group that had watched the video depicting the negative consequences of alcohol consumption, compared to the control group.

The visual probe task revealed no evidence of attentional bias or attentional avoidance of alcohol cues in any group (or in the sample as a whole), therefore our hypotheses regarding the influence of the videos on attentional bias can be rejected. One interpretation is that attentional bias is insensitive to experimental manipulations of the motivation to drink or to avoid alcohol, although the absence of attentional bias in the control group argues against this interpretation. In the next study we repeated the general methodology of study 1 so that we were again able to investigate the effects of the different videos on self-reported approach and avoidance of alcohol. Given the null results from the visual probe task, we omitted this task and replaced it with a measure of automatic approach and avoidance tendencies for alcohol cues.

## Study 2

In the alcohol version of the stimulus-response compatibility (SRC) task (Field et al., 2008), a manikin is presented on a computer screen either above or below an alcohol-related or neutral picture. Participants must move the manikin toward or away from the pictures as quickly as possible. On some blocks of the task, participants must make the manikin move toward alcohol pictures and away from neutral pictures, whereas these instructions are reversed in other blocks of the task. Automatic approach tendencies for alcohol cues are inferred if participants are faster to respond on blocks of the task when alcohol pictures require the “approach” movement in comparison to blocks when alcohol pictures require the “avoidance” movement. By contrast, if participants are faster on the “avoid alcohol” blocks compared to the “approach alcohol” blocks, this would suggest that alcohol cues evoke automatic avoidance tendencies. Heavy drinkers who are not seeking treatment display automatic approach tendencies for alcohol cues (Field et al., 2008, 2011; Christiansen et al., 2012; Kersbergen et al., 2015), whereas alcohol-dependent patients may show the opposite pattern, i.e., they are faster to avoid rather than approach alcohol-related pictures (Spruyt et al., 2013; but see Barkby et al., 2012).

Findings obtained from the standard version of the SRC task must be interpreted cautiously because this task yields an index of automatic approach that is *relative* to automatic avoidance, therefore an apparent bias in automatic approach could be attributed to either strong automatic approach, weak automatic avoidance, or a combination of the two. Among alcohol-dependent patients, if alcohol cues simultaneously evoke strong automatic approach at the same time as strong automatic avoidance, this may explain why they display either no reliable bias on the task (Barkby et al., 2012) or a bias to faster avoidance (Spruyt et al., 2013) depending on the strength of their automatic tendencies to avoid alcohol at the time of testing. In the present study, we overcame this limitation by modifying the task so that it is able to distinguish automatic alcohol approach and avoidance tendencies from each other. This modified version of the task includes neutral movements (to the side) in addition to the standard approach and avoidance movements, and is split into four blocks instead of two (see Baker et al., 2014).

The present study was identical to study 1 with the important difference that participants completed a modified SRC task instead of the visual probe task that was used in study 1. We hypothesized that we would replicate the effects of the videos on the AAAQ that were observed in study 1. Regarding the indices of approach and avoidance tendencies from the SRC task, we hypothesized that, relative to the control group, participants in the alcohol-positive group would show stronger automatic alcohol approach tendencies but these groups would not differ in automatic avoidance tendencies. By contrast, relative to the control group we anticipated stronger automatic avoidance tendencies in the alcohol-negative group, but these two groups would not differ in automatic approach tendencies.

### Methods

#### Participants

Ninety participants (56 Female, mean age 24.56, *SD* = 5.34) were recruited from the local community and students and staff at the University of Liverpool via online and poster advertising. Inclusion and exclusion criteria were identical to those described for study 1. Participants who had taken part in studies 1 or 3 were ineligible to participate. Participants provided informed consent before taking part in the study, which was approved by the University of Liverpool Research Ethics Committee.

#### Materials

##### The modified stimulus-response compatibility task

The modified stimulus-response compatibility Task (Baker et al., 2014) is used to measure automatic approach and avoidance responses evoked by alcohol-related cues. Participants are instructed to rapidly categorize alcohol-related and stationery-related (control) pictures by moving a manikin either toward or away from the pictures, or to the left (neutral movement), as quickly as possible by pressing one of three specific keys on the keyboard, which were labeled with arrows pointing up, down, and left. The task was programmed in Inquisit software (Millisecond Software, 2006) and presented on a laptop computer with a 13 inch screen.

The format of the task, trial structure, and perceptual characteristics of the pictorial stimuli were identical to those used in previous studies (Field et al., 2011; Barkby et al., 2012). Fourteen colored pictures (a subset of the picture set used in Barkby et al., 2012) were used in the task: seven pictures of alcoholic drinks and close-ups of individuals holding or consuming those drinks, and seven control pictures of stationery items and close-ups of models interacting with those items.

There were four sub-blocks of the task, which differed according to task instructions. In the “approach alcohol” block, participants were required to move the manikin toward alcohol pictures, and to the left for stationery pictures. In the “avoid alcohol” block, participants moved away from alcohol pictures and to the left for stationery pictures. In the “approach control” block, participants moved toward stationery pictures and to the left for alcohol pictures. Finally, in the “avoid control” block, participants moved away from stationery pictures and to the left for alcohol pictures. Note that in the case of approach and avoidance movements, the position of the manikin was crucial: if the manikin was above the picture, an “approach” response required participants to press the “down” key, and an “avoidance” response required participants to press the “up” key. This was reversed if the manikin was below the picture. Participants were instructed to respond quickly and accurately on each trial. If they pressed the correct key, the manikin moved up, down or to the left in an animation lasting 500 ms. If they pressed the wrong key, error feedback was provided in the form of a large red cross presented in the center of the screen for 500 ms. There was an inter-trial interval of 500 ms.

Each sub-block of the task comprised four practice trials, in which two alcohol pictures and two control pictures were presented, once with the manikin above each picture type and once with the manikin below. If participants did not understand the task, this practice block was repeated. There then followed 28 “critical” trials, in which each of the 14 pictures was presented twice: once with the manikin above the picture and once with the manikin below. Trials were presented in a new random order for each participant. Participants completed the sub-blocks in a counterbalanced order. Responses and reaction times (in milliseconds) to initiate the manikin movement were recorded on each trial.

#### Procedure

Participants were randomly allocated to experimental conditions. They were tested in a laboratory in the Department of Psychological Sciences or in quiet public places in which alcohol was not available (e.g., cafes and libraries). After providing informed consent, participants completed the timeline followback drinking diary, AUDIT, AAAQ, and PANAS (time 1). Then, participants put on the headphones and watched one of the videos, before completing the Video Questionnaire and the AAAQ and PANAS again (time 2). Finally, participants completed the SRC task. After completing the study, participants were debriefed and offered either course credit or a £5 Shopping Voucher to compensate them for their time.

### Results

#### Group characteristics

Participants reported consuming 30.21 (*SD* = 23.53) units of alcohol per week, and the mean score on the AUDIT was 12.85 (*SD* = 5.34). There were no between-group differences in age, weekly alcohol consumption, or AUDIT scores (all Kruskal–Wallis tests *p* > 0.1). There were no group differences in gender ratio (χ^2^ = 0.66, *p* > 0.1).

#### Effects of video manipulation on AAAQ ratings (Figure 1B)

AAAQ ratings were analyzed using a mixed design ANOVA, with within-subject factors of sub-scale (3: inclined-indulgent, obsessed-compelled, resolved-regulated), time (2: before video, after video), and group (3: alcohol-positive, alcohol-negative, control). The sub-scale × time × group interaction was statistically significant [*F*_(4, 174)_ = 19.16, *p* < 0.001]. Subsequent *post-hoc* ANOVAs confirmed that the time x group interaction was significant for all three sub-scales [inclined-indulgent *F*_(2, 87)_ = 19.67, *p* < 0.001]; obsessed-compelled *F*_(2, 87)_ = 5.28, *p* < 0.01; resolved-regulated *F*_(2, 87)_ = 13.80, *p* < 0.001].

Groups did not differ on the inclined-indulgent [*F*_(2, 89)_ = 1.56, *p* > 0.1] or obsessed-compelled [*F*_(2, 89)_ = 1.26, *p* > 0.1] sub-scales before watching the video. However, there was a group difference in the resolved-regulated sub-scale before the video [*F*_(2, 89)_ = 3.90, *p* < 0.05], and *post-hoc* LSD contrasts revealed that scores were lower in the control group compared to both the alcohol-positive and alcohol-negative groups (*p* < 0.05), who did not differ from each other (*ps* > 0.1). As predicted, groups differed on all three sub-scales after watching the video [inclined-indulgent *F*_(2, 89)_ = 10.78, *p* < 0.001; obsessed-compelled *F*_(2, 89)_ = 4.85, *p* = 0.01; resolved-regulated *F*_(2, 89)_ = 20.78, *p* < 0.001]. *Post-hoc* LSD contrasts revealed that scores on both inclined-indulgent and obsessed-compelled sub-scales were higher in the alcohol-positive group compared to both alcohol-negative and control groups (*p* < 0.01), who did not differ from each other (*p* > 0.1). On the other hand, scores on the resolved-regulated sub-scale were higher in the alcohol-negative group compared to both alcohol-positive and neutral groups (*ps* < 0.01), who did not differ from each other (*p* > 0.08).

Paired-samples *t*-tests revealed that among participants in the alcohol positive group, scores on the inclined-indulgent and obsessed-compelled sub-scales increased after watching the video [*t*_(29)_ = 2.74, *p* = 0.01 and *t*_(29)_ = 2.84, *p* < 0.01], whereas scores on the resolved-regulated sub-scale decreased [*t*_(29)_ = 2.90, *p* < 0.01]. The reverse pattern was seen in the alcohol negative group: the decrease in inclined-indulgent ratings and the increase in resolved-regulated ratings after watching the video were statistically significant [*t*_(29)_ = 6.70, *p* < 0.001 and *t*_(29)_ = 3.15, *p* < 0.01], although there was no significant change in scores on the obsessed-compelled sub-scale [*t*_(29)_ = 0.32, *p* > 0.1]. In the control group, scores on both the inclined-indulgent and resolved-regulated sub-scales decreased after watching the video [*t*_(29)_ = 2.56, *p* < 0.05 and *t*_(29)_ = 2.63, *p* < 0.05], but scores on the obsessed-compelled sub-scale did not change [*t*_(29)_ = 0.27, *p* > 0.1].

We also re-ran the omnibus Three-Way ANOVA on AAAQ scores but added PANAS positive and PANAS negative affect after the video as covariates. The three way interaction sub-scale × time × group remained statistically significant [*F*_(4, 170)_ = 9.55, *p* < 0.001]. Therefore, statistically controlling for positive and negative mood at the time did not influence the influence of the videos on the AAAQ.

#### SRC task (Table 2)

Data were analyzed in accordance with previous studies (e.g., Field et al., 2011). Firstly, trials with errors were discarded, and then outlying reaction times were removed if they were faster than 200 ms, slower than 2000 ms, and then if they were more than three standard deviations above the individual mean. All data from three participants were excluded as they had an outlying high rate (> 40%) of missing data due to errors and outliers. For the remainder of the sample, on average 5% of trials were missing due to errors and a further 9% due to outliers, and these values did not differ between groups (*ps* > 0.1).

**Table 2:** **Mean reaction times (in milliseconds) from the different blocks of the SRC task in study 2**.

	**Alcohol-positive**	**Alcohol-negative**	**Control**
Approach alcohol	826.51 ± 165.05	855.10 ± 186.92	871.38 ± 208.88
Approach stationery	855.99 ± 198.96	878.74 ± 186.11	917.37 ± 195.66
Avoid alcohol	835.32 ± 133.79	893.49 ± 207.91	895.22 ± 208.31
Avoid stationery	878.81 ± 174.65	918.22 ± 202.01	902.41 ± 211.75

*Values are means ± SD*.

Mean reaction times in the different blocks of the task were then analyzed using a mixed design 2 × 2 × 3 ANOVA, with within-subject factors of movement type (2: approach, avoidance) and picture type (2: alcohol, stationery; this refers to the type of picture that the approach or avoidance movement had to be directed toward or away from, with the sideways movement required for the other type of picture), and a between-subjects factor of group. The hypothesized three way interaction was not statistically significant [*F*_(2, 84)_ = 0.80, *p* > 0.1]. There were, however, significant main effects of picture type [*F*_(1, 84)_ = 4.75, *p* < 0.05; participants were faster to respond on blocks when the approach or avoidance movement had to be made in response to alcohol pictures rather than stationery pictures], and movement type [*F*_(1, 84)_ = 4.82, *p* < 0.05; participants were faster on “approach” blocks than “avoid” blocks of the task). The picture type x movement type interaction was not statistically significant [*F*_(1, 84)_ = 0.19, *p* > 0.1]. Overall, these results show that participants were faster to make approach rather than avoidance movements, and they were faster to make both approach and avoidance movements in response to alcohol pictures in comparison to stationery pictures. However, the video manipulation had no effect on performance on the task.

### Discussion

Consistent with results from study 1, results from this study did not provide clear support for the independence of self-reported approach and avoidance inclinations for alcohol following an experimental manipulation of those inclinations. Between-group contrasts demonstrated that, relative to the control group, scores on the inclined-indulgent and obsessed-compelled subscales of the AAAQ were elevated in participants who had watched a video depicting the positive consequences of alcohol consumption, but this video did not influence scores on the resolved-regulated subscale. The complete opposite pattern was seen in participants who had watched a video depicting the negative consequences of alcohol consumption: increased scores on the resolved-regulated subscale, but there was no difference between this group and the control group in scores on the inclined-indulgent and obsessed-compelled subscales. These contrasts support predictions made by the ambivalence model of craving (Breiner et al., 1999), because they suggest that it is possible to experimentally manipulate subjective approach and avoidance inclinations for alcohol independently of each other. Unfortunately, this conclusion must be heavily caveated given the presence of group differences on the resolved-regulated subscale at baseline, and because within-subject contrasts suggest that increases in approach inclinations were accompanied by decreases in avoidance inclinations in the alcohol positive group, and vice versa for the alcohol negative group.

The data from the SRC task also did not support our hypotheses: there was no evidence that participants were faster to approach or slower to avoid alcohol cues compared to stationery (control) cues, and the experimental manipulation did not influence the task. Therefore, even though our experimental manipulation had a clear influence on self-reported approach and avoidance inclinations for alcohol, there were no parallel changes in automatic approach or avoidance tendencies evoked by alcohol cues. In our third and final study, we again investigated the influence of alcohol-positive and alcohol-negative videos on self-reported (AAAQ) and automatic (modified SRC task) indices of approach and avoidance motivational orientations for alcohol, but we combined this with an experimental manipulation of thought suppression in an attempt to maximize the influence of the video manipulation on automatic measures of approach and avoidance.

## Study 3

People who are attempting to reduce their alcohol consumption often attempt to suppress unwanted thoughts, such as intrusive cravings, in order to achieve their goal (Moss et al., 2015). Unfortunately, thought suppression has unwelcome consequences because it increases, rather than decreases the frequency of intrusive thoughts that are the target of suppression, but only when competing demands are placed on cognitive resources (Wenzlaff and Wegner, 2000). Indeed, attempting to suppress thoughts about alcohol paradoxically increases the accessibility of alcohol-related cognitions as evidenced by increased attentional bias for alcohol words (Klein, 2007) and accessibility of alcohol-related semantic associations (Palfai et al., 1997).

We hypothesized that if participants were primed to think about the positive or negative consequences of alcohol consumption and were then instructed to suppress those thoughts, this should provoke an increase in the accessibility of those thoughts that would manifest itself as a bias in automatic approach or avoidance tendencies in response to alcohol cues. Specifically, participants who viewed a video depicting the positive consequences of alcohol and then attempted to suppress thoughts about alcohol should have stronger automatic approach tendencies for alcohol cues compared to participants who watched the same video but did not attempt to suppress their thoughts. We expected comparable moderating effects of thought suppression on automatic avoidance tendencies in participants who watched a video depicting the negative consequences of alcohol.

### Methods

#### Participants

One hundred participants (53 Female, mean age 27.87, *SD* = 6.97) were recruited from the local community and students and staff at the University of Liverpool via online and poster advertising. Inclusion and exclusion criteria were identical to those described for studies 1 and 2. Participants who had taken part in studies 1 or 2 were ineligible to participate. All participants provided informed consent before taking part in the study, which was approved by the University of Liverpool Research Ethics Committee.

#### Procedure

Participants were randomly allocated to one of four experimental conditions: (1) alcohol-positive video combined with thought suppression, (2) alcohol-positive video combined with control manipulation, (3) alcohol-negative video combined with thought suppression, or (4) alcohol-negative video combined with control manipulation. Note that none of the participants in this study watched the neutral video that we used in studies 1 and 2. Participants were tested in a laboratory in the Department of Psychological Sciences or in quiet public places in which alcohol was not served (e.g., cafes, libraries). After providing informed consent, participants completed the timeline follow back drinking diary, AUDIT, AAAQ, and PANAS (time 1). Then, participants put on the headphones and watched one of the videos, before completing the Video Questionnaire and the AAAQ and PANAS again (time 2).

Participants in the thought suppression groups were then instructed to think about anything, but to make every effort to suppress thoughts of alcohol; the importance of the latter was emphasized. Participants in the control groups were instructed to think about anything that came to mind, including alcohol. All participants were then given a 5 min to think freely and to write notes about what they were thinking about on a piece of paper. They were also instructed to place a mark in the right hand margin of the paper each time they thought about alcohol; these marks were subsequently counted up and cross-checked with the content of participants' notes.

Immediately after this 5-min period, participants were asked to respond to two questions by placing a mark on 100 ms visual analog scales (VAS). The questions, which were the same for both groups, were: “To what extent did you think about alcohol”? (anchors “I did not think about alcohol at all” and “I thought about alcohol a lot”) and “To what extent did you succeed in complying with the instructions” (anchors “totally unsuccessful” and “totally successful”).

Participants then completed the SRC task. The thought suppression or control instructions were re-iterated to participants before each block of the task. In order to increase demands on working memory, participants were given a seven-digit number at the beginning of each sub-block of the task. They were given 50 s to memorize the number and then instructed to hold it in memory, as they would be asked to recall it at the end of each sub-block of the task. Recall of this number was recorded at the end of each sub-block and the process was repeated with a different number at the beginning of each sub-block (see Bryant et al., 2011). After completing all blocks of the SRC task, participants again completed the two 100 ms VAS to indicate the extent to which they had thought about alcohol, and had complied with instructions, whilst they were doing the task. Finally, participants were debriefed and offered either course credit or a £5 Shopping Voucher to compensate them for their time.

### Results

#### Group characteristics

Participants reported consuming 22.53 (*SD* = 15.63) units of alcohol per week, and the mean score on the AUDIT was 10.56 (*SD* = 4.52). There was no group difference in gender ratio (χ^2^ = 4.62, *p* > 0.1), although there were group differences in both weekly alcohol consumption and AUDIT scores (Kruskal–Wallis tests, *ps* < 0.05). Participants in both thought suppression groups had higher weekly alcohol consumption and higher scores on the AUDIT compared to participants in both control groups. Therefore, we repeated all primary analyses (detailed below) with the addition of weekly alcohol consumption and AUDIT scores as covariates.

#### Effects of video manipulation on AAAQ ratings (Figure 1C)

AAAQ ratings were analyzed using a mixed design ANOVA, with within-subject factors of sub-scale (3: inclined-indulgent, obsessed-compelled, resolved-regulated), time (2: before video, after video), and between-subject factors of video group (2: alcohol-positive, alcohol-negative), and thought suppression group (2: thought suppression, control). The sub-scale x time x video group interaction was statistically significant [*F*_(2, 95)_ = 41.60, *p* < 0.001] but the four way interaction sub-scale × time × video group × thought suppression group was not [*F*_(2, 95)_ = 1.17, *p* > 0.1]. The three way interaction remained significant after adding AUDIT scores and weekly alcohol consumption as covariates [*F*_(2, 93)_ = 38.40, *p* < 0.001]. Subsequent *post-hoc* ANOVAs confirmed that the time x video group interaction was significant for all three sub-scales [inclined-indulgent *F*_(1, 98)_ = 40.51, *p* < 0.001; obsessed-compelled *F*_(1, 98)_ = 23.84, *p* < 0.001; resolved-regulated *F*_(1, 98)_ = 34.35, *p* < 0.001].

Groups did not differ on any of the sub-scales before watching the video [inclined-indulgent *t*_(98)_ = 0.08, *p* > 0.1; obsessed-compelled *t*_(98)_ = 1.62, *p* > 0.1; resolved-regulated *t*_(98)_ = 1.46, *p* > 0.1]. As predicted, groups differed on all three sub-scales after watching the video [inclined-indulgent *t*_(98)_ = 4.06, *p* < 0.001, higher in the alcohol-positive video group; obsessed-compelled *t*_(98)_ = 2.15, *p* < 0.05, also higher in the alcohol-positive video group; resolved-regulated *t*_(98)_ = 5.68, *p* < 0.001, higher in the alcohol-negative video group].

Paired-samples *t*-tests revealed that in the alcohol-positive video group, scores on the inclined-indulgent and obsessed-compelled sub-scales increased after watching the video [*t*_(49)_ = 1.96, *p* < 0.05 and *t*_(49)_ = 3.12, *p* < 0.01), whereas scores on the resolved-regulated sub-scale decreased [*t*_(49)_ = 2.20, *p* < 0.05]. The reverse pattern was seen in the alcohol-negative group: scores on the inclined-indulgent and obsessed-compelled sub-scales decreased after watching the video [*t*_(49)_ = 6.16, *p* < 0.001 and *t*_(49)_ = 3.95, *p* < 0.001] whereas scores on the resolved-regulated sub-scale increased [*t*_(49)_ = 5.45, *p* < 0.001].

We also re-ran the omnibus Four-Way ANOVA on AAAQ scores but added PANAS positive and PANAS negative affect after the video as covariates. The three way interaction sub-scale x time x group remained statistically significant [*F*_(2, 93)_ = 30.79, *p* < 0.001]. Therefore, statistically controlling for positive and negative mood at the time did not influence the influence of the videos on the AAAQ.

#### SRC task (Table 3)

All data were missing from two participants due to an experimenter error. Data were analyzed as described for study 2. All data from two additional participants were excluded as they had an outlying high rate (> 35%) of missing data due to errors and outliers. For the remaining participants, on average 4% of trials were missing due to errors and a further 7% due to outliers; these values did not differ between groups (*ps* > 0.1).

**Table 3:** **Mean reaction times (in milliseconds) from the different blocks of the SRC task in study 3**.

	**Suppress positive**	**Suppress negative**	**Control positive**	**Control negative**
Approach alcohol	905.47 ± 256.62	815.36 ± 242.46	943.52 ± 261.85	871.95 ± 244.25
Approach stationery	1018.24 ± 308.49	877.71 ± 236.56	960.42 ± 234.93	928.08 ± 289.12
Avoid alcohol	934.29 ± 219.92	842.58 ± 238.03	954.69 ± 207.22	882.97 ± 223.72
Avoid stationery	936.15 ± 244.13	871.00 ± 234.06	941.56 ± 207.04	915.88 ± 249.54

*Values are means ± SD*.

Mean reaction times in the different blocks of the task were then analyzed using a mixed design 2 × 2 × 2 × 2 ANOVA, with within-subject factors of movement type (2: approach, avoidance) and picture type (2: alcohol, stationery; this refers to the type of picture that the approach or avoidance movement had to be directed toward or away from, with the sideways movement required for the other type of picture), and between-subject factors of video group (2: alcohol-positive, alcohol-negative), and thought suppression group (2: thought suppression, control). The hypothesized four way interaction was not statistically significant [*F*_(1, 92)_ = 0.99, *p* > 0.1]. There was, however, a significant main effect of picture type [*F*_(1, 92)_ = 11.22, *p* < 0.01] which was subsumed under a significant picture type x movement type interaction [*F*_(1, 92)_ = 7.85, *p* < 0.01]. Paired-samples *t*-tests revealed that participants were significantly faster to approach alcohol rather than control pictures [*t*_(95)_ = 4.45, *p* < 0.001], but reaction times to avoid alcohol and control pictures did not differ [*t*_(95)_ = 0.97, *p* > 0.1].

There were no other significant main effects or interactions (*F*s < 1.68, *ps* > 0.1), and results were unaffected when the analysis was repeated with AUDIT scores and weekly alcohol consumption added as covariates. Overall, these results show that participants were faster to make approach movements to alcohol pictures than control pictures, but there was no difference in the speed of avoidance movements. Most importantly, neither the video manipulation or the thought suppression manipulation, or the interaction between the two, had any effect on performance on the task.

#### Thought suppression and working memory load manipulation checks

Responses on the visual analog scales, and the number of alcohol-related thoughts that participants recorded, are shown in Table 4. Each VAS was analyzed using a separate 2 × 2 ANOVA, with between-subject factors of video group (2: alcohol-positive, alcohol-negative), and thought suppression group (2: thought suppression, control). There were no main effects or interactions for the “To what extent did you succeed in complying with the instructions”? VAS at either time (*Fs* < 2.99, *p*s > 0.08). There were no main effects or interactions for the “To what extent did you think about alcohol” question immediately after the thought suppression and thought listing exercise (*Fs* < 2.24, *ps* > 0.1), which suggests that the thought suppression manipulation was not effective. However, the main effect of thought suppression group was statistically significant for this question immediately after participants had completed the SRC task [*F*_(1, 99)_ = 8.74, *p* < 0.001], as participants in the thought suppression group reported significantly fewer alcohol-related thoughts while completing the SRC task than participants in the control group. There were no other main effects or interactions (*Fs* < 1.80, *ps* > 0.1). Finally, there were no significant main effects or interactions for the number of alcohol-related thoughts that participants recorded during the thought suppression and thought listing exercise (*Fs* < 0.72, *ps* > 0.1).

**Table 4:** **The effect of thought suppression on self-reported alcohol-related thoughts and task instructions**.

	**Suppress positive**	**Suppress negative**	**Control positive**	**Control positive**
**IMMEDIATELY AFTER THOUGHT SUPPRESSION AND THOUGHT LISTING EXERCISE:**				
“To what extent did you think about alcohol”?	30.92 ± 27.23	37.96 ± 31.43	43.60 ± 32.34	43.60 ± 31.08
“To what extent did you succeed in complying with instructions”?	67.48 ± 26.71	59.76 ± 32.48	74.60 ± 22.63	71.36 ± 25.57
Number of alcohol-related thoughts recorded	3.92 ± 3.92	4.68 ± 3.81	4.32 ± 2.82	4.76 ± 3.50
**IMMEDIATELY AFTER COMPLETING SRC TASK:**				
“To what extent did you think about alcohol”?	27.64 ± 26.23	18.16 ± 20.45	36.72 ± 32.61	42.32 ± 31.51
“To what extent did you succeed in complying with instructions”?	75.36 ± 15.16	75.76 ± 25.08	78.72 ± 21.35	74.88 ± 21.24

*Visual analog scales are 100 ms VAS. Values are means ± SD*.

Overall, these results indicate that the thought suppression manipulation was not successful, because there were no differences in perceived suppression success or the number of alcohol-related thoughts recorded between thought suppression and control groups during the thought listing exercise. However, when asked immediately after completing the SRC task, participants in the thought suppression group reported that they thought about alcohol significantly *less* than participants in the control group. Furthermore, the lack of significant thought suppression x video group interactions for these measures suggests that the alcohol positive and alcohol negative videos did not have differential effects on the success of attempted thought suppression.

Finally, 97% of participants successfully recalled the 7-digit number at the end of each sub-block of the SRC task, which demonstrates that compliance with the manipulation of working memory load was very high.

### Discussion

Consistent with the findings from studies 1 and 2, results from this study demonstrated that participants who watched a video depicting the positive consequences of alcohol reported an increase in approach inclinations for alcohol that was accompanied by a reduction in avoidance inclinations; the converse pattern was seen among participants who watched a video depicting the negative consequences of alcohol consumption.

The primary novel feature of this study was the incorporation of a thought suppression manipulation in an attempt to magnify the influence of the videos on automatic approach and avoidance responses evoked by alcohol-related cues. Contrary to our hypotheses, we observed no evidence that the thought suppression manipulation led to an increase in alcohol-related thoughts; by contrast, participants' self-reports indicated that they were able to suppress alcohol when instructed to do so. We observed that participants were faster to approach alcohol rather than control pictures, thereby replicating previous findings using a related task (Field et al., 2008, 2011; Christiansen et al., 2012; Kersbergen et al., 2015). However, this pattern of results was unaffected by the videos, the thought suppression manipulation, or the interaction between the two.

## Combined analysis

Results from all three studies demonstrated that self-reported inclinations to approach and avoid alcohol were not independent of each other: increases in approach inclinations were accompanied by parallel decreases in avoidance inclinations, and vice versa. This interpretation could be bolstered by investigating the strength of the associations between *changes* in subjective approach and avoidance inclinations for alcohol after exposure to videos depicting the positive and negative consequences of alcohol consumption.

To this end, we combined the AAAQ data from all three studies, but disregarded data from the control groups (participants who watched the neutral video) in studies 1 and 2. Given that the thought suppression manipulation had no influence on the AAAQ in study 3, we collapsed these data across thought suppression groups. This combined analysis, with a total sample size of 221, confirmed that the sub-scale × time × group interaction was highly statistically significant, and that the time × group interactions were highly significant for all three of the AAAQ subscales (all *ps* < 0.001). Furthermore, paired samples *t*-tests confirmed that in the combined alcohol-positive group, scores on the inclined-indulgent and obsessed-compelled subscales increased, whereas scores on the resolved-regulated subscale decreased, after watching the video. The reverse pattern was seen in the combined alcohol-negative group. Data are shown in Table S4.

We then computed change scores to capture the change in each AAAQ subscale after participants watched the videos. By correlating these change scores with each other we were able to investigate the strength of the association between changes in self-reported approach and avoidance inclinations: as approach inclinations increase, do avoidance inclinations decrease by a similar magnitude (and vice versa)? Overall, intercorrelations between these change scores were statistically significant but small. After watching a video depicting the positive consequences of alcohol consumption, the magnitude of the increase in scores on the inclined-indulgent and obsessed-compelled subscales was associated with the magnitude of the decrease in scores on the resolved-regulated subscale, although the size of the correlation co-efficients (*r* = −0.20) suggests only 4% shared variance. After watching a video depicting the negative consequences of alcohol consumption, the magnitude of the increase in scores on the resolved-regulated subscale was weakly associated with the reduction in scores on the inclined-indulgent subscale (*r* = −0.36; 13% shared variance), but was unrelated to the reduction in scores on the obsessed-compelled subscale.

Finally, we are grateful to the reviewer who suggested the following additional analyses, which are detailed in Table S5. We performed hierarchical linear regression analyses to investigate if the change score for self-reported approach inclinations would be predicted by the video manipulation, even after entering the change score for self-reported avoidance inclinations in the first step of the model. With the score on the inclined-indulgent subscale as the dependent variable, the score on the resolved-regulated subscale accounted for 22% of variance [*F*_(1, 279)_ = 77.29, *p* < 0.01], but addition of experimental group as a predictor in the subsequent step of the regression accounted for an additional 8% of variance [*FΔ*_(1, 277)._ = 29.73, *p* < 0.01]. Similarly, with the score on the obsessed-compelled subscale as the dependent variable, the score on the resolved-regulated subscale accounted for 7% of variance [*F*_(1, 279)_ = 22.20, *p* < 0.01], but addition of experimental group as a predictor accounted for an additional 6% of variance [*FΔ*_(1, 277)._ = 18.42, *p* < 0.01].

However, we did not observe the same pattern when scores on the resolved-regulated subscale were entered as the dependent variable, and scores on the inclined-indulgent and obsessed-compelled subscales were entered as independent variables (in separate analyses). In the first case, after accounting for the 22% of variance attributable to the inclined-indulgent subscale [*F*_(1, 279)_ = 77.29, *p* < 0.01], the addition of experimental group as a predictor did not account for additional variance in scores on the resolved-regulated subscale [ < 1% of additional variance; *FΔ*_(1, 277)._ = 1.07, *p* = 0.30]. Similarly, after accounting for the 7% of variance attributable to the obsessed-compelled subscale [*F*_(1, 279)_ = 22.20, *p* < 0.01], the addition of experimental group as a predictor did not account for additional variance in scores on the resolved-regulated subscale [ < 1% of additional variance; *FΔ*_(1, 277)._ = 0.20, *p* = 0.66].

These analyses reveal that self-reported approach and avoidance inclinations for alcohol can operate at least partly independently of each other, but the effect is not symmetrical. Changes in self-reported avoidance inclinations after watching videos depicting the positive or negative consequences of alcohol consumption were completely accounted for by changes in self-reported approach inclinations. However, the changes in self-reported approach inclinations that were evoked by these videos were at least partly independent of changes in self-reported avoidance inclinations.

## General discussion

A number of consistent findings emerged from the three studies reported here. When participants viewed short videos that depicted the positive or negative consequences of alcohol consumption, their self-reported approach and avoidance inclinations for alcohol tended to change in parallel: as approach inclinations increased, avoidance inclinations decreased, and vice versa. However, between-group contrasts suggested some degree of independence of approach and avoidance inclinations, although these findings were not consistent across studies. In addition, although *changes* in approach and avoidance inclinations tended to be inversely correlated (as one increased, the other decreased), these correlations were small and there was some evidence that changes in approach inclinations were partly independent of changes in avoidance inclinations. Finally, we found no evidence in support of our predictions that alcohol-related implicit cognitions would be influenced by experimental manipulations of motivational orientations for alcohol, regardless of whether we measured attentional biases or automatic approach / avoidance tendencies, or whether the experimental manipulation was combined with a thought suppression exercise.

One of the primary aims of these studies was to expand on findings from previous studies that used the Approach and Avoidance of Alcohol Questionnaire (AAAQ) in order to test predictions made by the ambivalence model of craving (Breiner et al., 1999). Specifically, if subjective approach and avoidance inclinations for alcohol are independent of each other, it should be possible to dissociate them by exposing participants to experimental manipulations that are designed to increase one but not the other. Overall, our findings demonstrated that approach and avoidance inclinations tend to change in parallel because as one increased, the other tended to decrease. This casts doubt on the independence of these constructs as predicted by the ambivalence model. Specifically, within-subject contrasts revealed that, after watching a video depicting the positive consequences of alcohol consumption, approach inclinations increased and avoidance inclinations decreased, whereas the reverse pattern was seen in participants who watched a video depicting the negative consequences of alcohol consumption. Although there were some minor inconsistencies between studies, the results of a combined analysis of data from all studies confirmed that approach and avoidance inclinations tended to fluctuate alongside each other. Furthermore, the combined analysis revealed that the magnitude of changes in approach and avoidance inclinations over time were reliably negatively correlated with each other: the magnitude of the increase in the strength of approach inclinations was associated with the magnitude of the corresponding decrease in avoidance inclinations, and vice versa. Finally, the regression analysis confirmed that variation in the change in avoidance inclinations after watching the videos was completely accounted for by the change in approach inclinations.

However, other analyses suggested that self-reported approach and avoidance inclinations could be characterized as at least partly independent of each other. Although the combined analysis confirmed inverse correlations between the magnitude of changes in approach and avoidance inclinations over time, these relationships were weak (with, at most, 13% shared variance), and regression analyses demonstrated that changes in approach inclinations were at least partly independent of changes in avoidance inclinations. More importantly, between-subject contrasts with a control group suggested that approach inclinations increased without a corresponding change in avoidance inclinations, and vice versa. Unfortunately, we have limited confidence in these group differences because they were only seen in study 2; in study 1, group differences in approach inclinations were accompanied by group differences in avoidance inclinations.

Our findings are consistent with some previous observations that subjective approach and avoidance inclinations for alcohol tend to change in parallel after exposure to appetitive alcohol cues (Curtin et al., 2005), although one study reported a dissociation between approach and avoidance inclinations after cue exposure (Jones et al., 2013). Importantly, the studies reported here are the very first to investigate the influence of an experimental manipulation that was intended to activate motivational orientations to avoid drinking; findings from participants in the alcohol-negative groups in all studies clearly suggest that this manipulation led to the predicted increase in self-reported avoidance inclinations that was accompanied by a decrease in approach inclinations. However, it is important to clarify that previous studies demonstrated that approach and avoidance inclinations have independent predictive validity for individual differences in drinking behavior and prospective drinking behavior (Curtin et al., 2005; Schlauch et al., 2012, 2013a,b,c; Klein and Anker, 2013). The three studies reported here cannot speak to the predictive validity of these constructs.

In contrast to the robust effects on self-reported approach and avoidance motivational orientations (assessed with the AAAQ), the videos depicting the positive and negative consequences of alcohol consumption had no effect on measures of motivational orientations operating within automatic processes, that is attentional biases (study 1) and approach / avoidance tendencies (studies 2 and 3). To our knowledge, these are the first studies that experimentally manipulated the motivation to avoid drinking and our findings suggest that these automatic processing biases are impervious to motivational orientations to avoid drinking. This interpretation is consistent with findings from an earlier study (Baker et al., 2014) in which we attempted to prime motivational orientations by presenting alcohol-positive and alcohol-negative primes below the threshold of conscious awareness; this manipulation also failed to influence attentional biases and approach / avoidance tendencies. However, on the basis of previous demonstrations of robust, albeit weak associations between subjective craving (typically assessed with self-report instruments that capture only “approach” inclinations) and attentional bias (Field et al., 2009), and demonstrations that experimental manipulations of craving such as negative mood induction, exposure to alcohol cues, and acute alcohol intoxication all lead to increases in attentional bias (see Field and Cox, 2008), we anticipated elevated attentional biases in the “attend positive” vs. the control groups. This pattern of results was not seen. Perhaps most importantly, with one exception (the bias to more rapidly approach alcohol rather than control images in study 3), we found no evidence of attentional or approach or avoidance biases in any of the studies, and no evidence that individual differences in alcohol consumption, hazardous drinking or scores on the AAAQ were associated with these implicit processing biases in any of the studies (see Supplementary Materials). These findings cast doubt on the validity and sensitivity of the tasks that were used in the current studies, all of which were slightly modified versions of tasks that are more commonly used in the literature. Future investigations of this research question should attempt to develop measures of approach and avoidance inclinations operating in automatic processes that have acceptable construct validity and sensitivity for this purpose.

The studies reported here have other weaknesses in addition to the questionable construct validity of the implicit measures. All participants consumed alcohol in excess of UK government guidelines and therefore their alcohol consumption was placing their health at risk. However, we did not attempt to recruit participants who were concerned about or attempting to limit their alcohol consumption, and we did not measure participants' motivation to change using a validated self-report measure, so it is possible that our participants were relatively insensitive to our experimental manipulations that were designed to exaggerate their ambivalence about alcohol consumption. Future studies could investigate this issue by recruiting heavy drinking participants who are currently motivated to reduce their alcohol consumption (and are actively attempting to do so), because motivational orientations in these participants might be expected to be more sensitive to the experimental manipulations that were used in the present study. A further limitation is that we did not record participants' occupational or socioeconomic status so we are unable to fully characterize participants who took part in these studies. In addition, it is possible that the videos had robust effects on self-report but not computerized measures because participants always completed the former before the latter; this could be investigated by counterbalancing the order in which assessments are administered in future studies. Our study also had strengths, including measurements of participants' subjective mood after they had watched the videos, which enabled us to rule out changes in mood as a contributor to the influence of those videos on self-reported and automatic motivational orientations.

In conclusion, findings from the three studies reported here question the degree of independence of self-reported approach and avoidance inclinations for alcohol, which tended to co-vary in response to experimental manipulations of inclinations to drink or inclinations to avoid alcohol. However, results from a combined analysis of data from all studies suggest that changes in inclinations to drink may be at least partially independent of changes in inclinations to avoid alcohol. Our findings also suggest that measures of alcohol-related motivational orientations that operate in automatic processes are impervious to these experimental manipulations, although the modified tasks that we used here have questionable construct validity and sensitivity which suggests that these findings should be interpreted with caution.

## Funding

Funded by a research grant from the Wellcome Trust, reference 086247/Z/08/Z, awarded to MF and JD.

### Conflict of interest statement

The authors declare that the research was conducted in the absence of any commercial or financial relationships that could be construed as a potential conflict of interest.

## References

[B1] Baker S. Dickson J. M. Field M. (2014). Implicit priming of conflicting motivational orientations in heavy drinkers. BMC Psychol. 2:28 10.1186/s40359-014-0028-1 PMC4591478 26483724

[B2] Barkby H. Dickson J. M. Roper L. Field M. (2012). To approach or avoid alcohol? Automatic and self-reported motivational tendencies in alcohol dependence. Alcohol. Clin. Exp. Res. 36, 361–368. 10.1111/j.1530-0277.2011.01620.x 21895719 PMC3417376

[B3] Breiner M. J. Stritzke W. G. K. Lang A. R. (1999). Approaching avoidance: a step essential to the understanding of craving. Alcohol. Res. Health 23, 197–206. 10890815 PMC6760377

[B4] Bryant R. A. Wyzenbeek M. Weinstein J. (2011). Dream rebound of suppressed emotional thoughts: the influence of cognitive load. Conscious. Cogn. 20, 515–522. 10.1016/j.concog.2010.11.004 21115260

[B5] Christiansen P. Cole J. C. Goudie A. J. Field M. (2012). Components of behavioural impulsivity and automatic cue approach predict unique variance in hazardous drinking. Psychopharmacology 219, 501–510. 10.1007/s00213-011-2396-z 21735071

[B6] Christiansen P. Schoenmakers T. M. Field M. (2015). Less than meets the eye: reappraising the clinical relevance of attentional bias in addiction. Addict. Behav. 44, 43–50. 10.1016/j.addbeh.2014.10.005 25453782

[B7] Curtin J. J. Barnett N. P. Colby S. M. Rohsenow D. J. Monti P. M. (2005). Cue reactivity in adolescents: measurement of separate approach and avoidance reactions. J. Stud. Alcohol 66, 332–343. 10.15288/jsa.2005.66.332 16047522

[B8] Dickson J. M. Gately C. Field M. (2013). Alcohol dependent patients have weak negative rather than strong positive implicit alcohol associations. Psychopharmacology 228, 603–610. 10.1007/s00213-013-3066-0 23503702 PMC3726926

[B9] Field M. Caren R. Fernie G. De Houwer J. (2011). Alcohol approach tendencies in heavy drinkers: comparison of effects in a relevant stimulus-response compatibility task and an approach/avoidance Simon Task. Psychol. Addict. Behav. 25, 697–701. 10.1037/a0023285 21534644

[B10] Field M. Cox W. M. (2008). Attentional bias in addictive behaviors: a review of its development, causes, and consequences. Drug Alcohol Depend. 97, 1–20. 10.1016/j.drugalcdep.2008.03.030 18479844

[B11] Field M. Kiernan A. Eastwood B. Child R. (2008). Rapid approach responses to alcohol cues in heavy drinkers. J. Behav. Ther. Exp. Psychiatry 39, 209–218. 10.1016/j.jbtep.2007.06.001 17640615

[B12] Field M. Mogg K. Bradley B. P. (2005). Craving and cognitive biases for alcohol cues in social drinkers. Alcohol Alcohol. 40, 504–510. 10.1093/alcalc/agh213 16157608

[B13] Field M. Mogg K. Mann B. Bennett G. A. Bradley B. P. (2013). Attentional biases in abstinent alcoholics and their association with craving. Psychol. Addict. Behav. 27, 71–80. 10.1037/a0029626 22905898

[B14] Field M. Mogg K. Zetteler J. Bradley B. P. (2004). Attentional biases for alcohol cues in heavy and light social drinkers: the roles of initial orienting and maintained attention. Psychopharmacology 176, 88–93. 10.1007/s00213-004-1855-1 15071718

[B15] Field M. Munafò M. R. Franken I. H. A. (2009). A Meta-Analytic investigation of the relationship between attentional bias and subjective craving in substance abuse. Psychol. Bull. 135, 589–607. 10.1037/a0015843 19586163 PMC2999821

[B16] Hettema J. Steele J. Miller W. R. (2005). Motivational interviewing. Annu. Rev. Clin. Psychol. 1, 91–111. 10.1146/annurev.clinpsy.1.102803.143833 17716083

[B17] Jones A. Rose A. K. Cole J. C. Field M. (2013). Effects of alcohol cues on craving and *ad libitum* alcohol consumption in social drinkers: the role of disinhibition. J. Exp. Psychopathol. 4, 239–240. 10.5127/jep.031912

[B18] Kersbergen I. Woud M. L. Field M. (2015). The validity of different measures of automatic alcohol action tendencies. Psychol. Addict. Behav. 29, 225–230. 10.1037/adb0000009 25134039

[B19] Klein A. A. Anker J. J. (2013). A psychometric evaluation of the approach and avoidance of alcohol questionnaire among alcohol-dependent patients attending residential treatment. J. Psychopathol. Behav. Assess. 35, 205–214. 10.1007/s10862-012-9322-5

[B20] Klein A. A. Stasiewicz P. R. Koutsky J. R. Bradizza C. M. Coffey S. F. (2007). A psychometric evaluation of the approach and avoidance of alcohol questionnaire (AAAQ) in alcohol dependent outpatients. J. Psychopathol. Behav. Assess. 29, 231–240. 10.1007/s10862-007-9044-2

[B21] Klein A. A. (2007). Suppression-induced hyperaccessibility of thoughts in abstinent alcoholics: A preliminary investigation. Behav. Res. Ther. 45, 169–177. 10.1016/j.brat.2005.12.012 16500617

[B22] Koster E. H. W. Crombez G. Verschuere B. De Houwer J. (2004). Selective attention to threat in the dot probe paradigm: differentiating vigilance and difficulty to disengage. Behav. Res. Ther. 42, 1183–1192. 10.1016/j.brat.2003.08.001 15350857

[B23] Lang P. J. Bradley M. M. Cuthbert B. N. (2008). International Affective Picture System (IAPS). Affective Ratings of Pictures and Instruction Manual. Technical Report A-8. Gainsville, FL: University of Florida.

[B24] Lee S. Cho S. Lee J. H. (2014). Approach-avoidance pattern of visual attention in hazardous drinkers with ambivalence. Addict. Behav. 39, 669–676. 10.1016/j.addbeh.2013.12.001 24368001

[B25] McEvoy P. M. Stritzke W. G. K. French D. J. Lang A. R. Ketterman R. L. (2004). Comparison of three models of alcohol craving in young adults: a cross-validation. Addiction 99, 482–497. 10.1111/j.1360-0443.2004.00714.x 15049748

[B26] Millisecond Software (2006). Inquisit (version 2). Seattle, WA: Millisecond Software.

[B27] Moss A. C. Erskine J. A. K. Albery I. P. Allen J. R. Georgiou G. J. (2015). To suppress, or not to suppress? That is repression: controlling intrusive thoughts in addictive behaviour. Addict. Behav. 44, 65–70. 10.1016/j.addbeh.2015.01.029 25648574

[B28] Noël X. Colmant M. Van Der Linden M. Bechara A. Bullens Q. Hanak C.. (2006). Time course of attention for alcohol cues in abstinent alcoholic patients: the role of initial orienting. Alcohol. Clin. Exp. Res. 30, 1871–1877. 10.1111/j.1530-0277.2006.00224.x 17067351

[B29] Palfai T. P. Monti P. M. Colby S. M. Rohsenow D. J. (1997). Effects of suppressing the urge to drink on the accessibility of alcohol outcome expectancies. Behav. Res. Ther. 35, 59–65. 10.1016/S0005-7967(96)00079-4 9009044

[B30] Peirce J. W. (2007). PsychoPy - Psychophysics software in Python. J. Neurosci. Methods 162, 8–13. 10.1016/j.jneumeth.2006.11.017 17254636 PMC2018741

[B31] Roefs A. Quaedackers L. Werrij M. Q. Wolters G. Havermans R. Nederkoorn C.. (2006). The environment influences whether high-fat foods are associated with palatable or with unhealthy. Behav. Res. Ther. 44, 715–736. 10.1016/j.brat.2005.05.007 16039602

[B32] Saunders J. B. Aasland O. G. Babor T. F. de la Fuente J. R. Grant M. (1993). Development of the alcohol use disorders identification test (AUDIT): WHO collaborative project on early detection of persons with harmful alcohol consumption II. Addiction 88, 791–804. 10.1111/j.1360-0443.1993.tb02093.x 8329970

[B33] Schlauch R. C. Breiner M. J. Stasiewicz P. R. Christensen R. L. Lang A. R. (2013a). Women inmate substance abusers' reactivity to visual alcohol, cigarette, marijuana, and crack-cocaine cues: approach and avoidance as separate dimensions of reactivity. J. Psychopathol. Behav. Assess. 35, 45–56. 10.1007/s10862-012-9313-6 23543075 PMC3608142

[B34] Schlauch R. C. Gwynn-Shapiro D. Stasiewicz P. R. Molnar D. S. Lang A. R. (2013b). Affect and craving: Positive and negative affect are differentially associated with approach and avoidance inclinations. Addict. Behav. 38, 1970–1979. 10.1016/j.addbeh.2012.12.003 23380493 PMC3578130

[B35] Schlauch R. C. Levitt A. Bradizza C. M. Stasiewicz P. R. Lucke J. F. Maisto S. A.. (2013c). Alcohol craving in patients diagnosed with a severe mental illness and alcohol use disorder: Bidirectional relationships between approach and avoidance inclinations and drinking. J. Consult. Clin. Psychol. 81, 1087–1099. 10.1037/a0033914 23895085 PMC3938894

[B36] Schlauch R. C. Stasiewicz P. R. Bradizza C. M. Coffey S. F. Gulliver S. B. Gudleski G. D. (2012). Relationship between approach and avoidance inclinations to use alcohol and treatment outcomes. Addict. Behav. 37, 824–830. 10.1016/j.addbeh.2012.03.010 22459327 PMC3356494

[B37] Sharbanee J. M. Stritzke W. G. K. Wiers R. W. Macleod C. (2013a). Alcohol-related biases in selective attention and action tendency make distinct contributions to dysregulated drinking behaviour. Addiction 108, 1758–1766. 10.1111/add.12256 23692442

[B38] Sharbanee J. M. Stritzke W. G. K. Wiers R. W. Young P. Rinck M. Macleod C. (2013b). The interaction of approach-alcohol action tendencies, working memory capacity, and current task goals predicts the inability to regulate drinking behavior. Psychol. Addict. Behav. 27, 649–661. 10.1037/a0029982 23088407

[B39] Sobell L. C. Sobell M. B. (1992). Timeline Follow-back: a technique for assessing self-reported ethanol consumption, in Measuring Alcohol Consumption: Psychosocial and Biological Methods, eds Allen J. Litten R. Z. (Totowa, NJ: Humana Press), 41–72. 10.1007/978-1-4612-0357-5_3

[B40] Spruyt A. De Houwer J. Tibboel H. Verschuere B. Crombez G. Verbanck P.. (2013). On the predictive validity of automatically activated approach/avoidance tendencies in abstaining alcohol-dependent patients. Drug Alcohol Depend. 127, 81–86. 10.1016/j.drugalcdep.2012.06.019 22776440

[B41] Stacy A. W. Wiers R. W. (2010). Implicit cognition and addiction: a tool for explaining paradoxical behavior. Annu. Rev. Clin. Psychol. 6, 551–575. 10.1146/annurev.clinpsy.121208.131444 20192786 PMC3423976

[B42] Stormark K. M. Field N. P. Hugdahl K. Horowitz M. (1997). Selective processing of visual alcohol cues in abstinent alcoholics: an approach-avoidance conflict? Addict. Behav. 22, 509–519. 10.1016/S0306-4603(96)00051-2 9290860

[B43] Townshend J. M. Duka T. (2001). Attentional bias associated with alcohol cues: differences between heavy and occasional social drinkers. Psychopharmacology 157, 67–74. 10.1007/s002130100764 11512045

[B44] Townshend J. M. Duka T. (2007). Avoidance of alcohol-related stimuli in alcohol-dependent inpatients. Alcohol. Clin. Exp. Res. 31, 1349–1357. 10.1111/j.1530-0277.2007.00429.x 17550367

[B45] Vollstädt-Klein S. Loeber S. Von Der Goltz C. Mann K. Kiefer F. (2009). Avoidance of alcohol-related stimuli increases during the early stage of abstinence in alcohol-dependent patients. Alcohol Alcohol. 44, 458–463. 10.1093/alcalc/agp056 19734158

[B46] Watson D. Clark L. A. Tellegen A. (1988). Development and validation of brief measures of positive and negative affect: the PANAS scales. J. Pers. Soc. Psychol. 54, 1063–1070. 10.1037/0022-3514.54.6.1063 3397865

[B47] Wenzlaff R. M. Wegner D. M. (2000). Thought suppression. Annu. Rev. Psychol. 51, 59–91. 10.1146/annurev.psych.51.1.59 10751965

[B48] Wiers R. W. Rinck M. Dictus M. van den Wildenberg E. (2009). Relatively strong automatic appetitive action-tendencies in male carriers of the OPRM1 G-allele. Genes Brain Behav. 8, 101–106. 10.1111/j.1601-183X.2008.00454.x 19016889

