# Supplementary material for: Priming of conflicting motivational orientations in heavy drinkers: robust effects on self-report but not implicit measures
Source: Front Psychol. 2015 Oct 2;6:1465. doi: 10.3389/fpsyg.2015.01465 (PMC4591478; doi:10.3389/fpsyg.2015.01465)
Supplement: Supplementary file 4 [file DataSheet1.DOCX]

Study 1

Effects of video manipulation on PANAS ratings (Table S1a)

PANAS ratings were analysed using a 2 x 2 x 3 mixed design ANOVA, with within-subject factors of sub-scale (2: positive, negative), time (2: before video, after video), and group (3: alcohol-positive, alcohol-negative, control). The three way interaction sub-scale x time x group was statistically significant (*F*(2, 87) = 8.92, *p* < .001). Subsequent post-hoc ANOVAs confirmed that the time x group interaction was significant for both the positive (*F*(2, 87) = 14.26, *p* < .001) and negative (*F*(2, 87) = 18.59, *p* < .001) sub-scales.

There were no group differences on either sub-scale before the video (positive *F*(2, 89) = 1.23, *p* > .1; negative *F*(2, 89) = .36, *p* > .1). However, group differences were apparent after the video (positive sub-scale *F*(2, 89) = 4.87, *p* < .001; negative sub-scale *F*(2, 89) = 13.32, *p* < .001). Post-hoc LSD (Least Significant Difference) contrasts confirmed that, after watching the video, PANAS positive sub-scale ratings were higher in the alcohol-positive group compared to the control group (*p* < .01), although the contrasts between alcohol-positive vs. alcohol-negative groups, and between alcohol-negative and control groups, were not significant (*ps* > .1). LSD contrasts on negative sub-scale ratings after the video revealed higher ratings in the alcohol-negative group compared to the alcohol-positive and control groups (*ps* < .01), although the latter groups did not differ from each other (*p* > .1).

We also performed within-subject t-tests, separately on each group, in order to identify changes in positive and negative affect that occurred as a result of watching the videos. In the alcohol-positive group, there was no change in either positive affect (*t*(28) = 1.01, *p* > .1), or negative affect (*t*(28) = 1.12, *p* > . 1) after watching the video. In the alcohol-negative group, positive affect did not change (*t*(31) = 1.58, *p* > .1), whereas negative affect increased (*t*(31) = 4.62, *p* < .001) after watching the video. In the control group, both positive affect (*t*(28) = 6.42, *p* < .01) and negative affect (*t*(29) = 3.68, *p* < .01) decreased after watching the video.

Effects of video manipulation on video questionnaire (Table S2a)

Participants’ responses were generally as anticipated – the alcohol-negative video was perceived as depicting the negative aspects of drinking, the alcohol-positive video was perceived as depicting the positive aspects of drinking, and the neutral video was perceived as boring and uninteresting. Each item was analysed using a separate one-way ANOVA, and each of these revealed a highly significant main effect of group (*Fs* > 74.45, *ps* < .001). Significant differences between conditions (LSD contrasts) are highlighted in the table.

Correlations with individual differences

We used non-parametric Spearman correlations to investigate the relationships between weekly alcohol consumption, AUDIT scores, scores on the three sub-scales of the AAAQ after watching the video, and attentional bias indices derived from the visual probe task (the mean RT on incongruent trials minus the mean RT on congruent trials, separately at each SOA). Scores on the AUDIT were positively correlated with scores on both the inclined-indulgent and obsessed-compelled sub-scales of the AAAQ (*rs* > .21, *ps*  < .05), whereas weekly alcohol consumption was only correlated with the inclined-indulgent sub-scale (*r* = .27, *p* < .01). Neither AUDIT scores nor weekly alcohol consumption were significantly correlated with scores on the resolved-regulated sub-scale (*ps* > .1), or with either of the attentional bias indices (*ps* > .1). Scores on the AAAQ were also unrelated to attentional biases (*ps* > .1).

Study 2

Effects of video manipulation on PANAS ratings (Table S1b)

*PANAS ratings* were analysed using a 2 x 2 x 3 mixed design ANOVA, with within-subject factors of sub-scale (2: positive, negative), time (2: before video, after video), and group (3: alcohol-positive, alcohol-negative, control). The three way interaction sub-scale x time x group was statistically significant (*F*(2, 87) = 11.54, *p* < .001). Subsequent post-hoc ANOVAs confirmed that the time x group interaction was significant for both the positive (*F*(2, 87) = 13.77, *p* < .001) and negative (*F*(2, 87) = 24.50, *p* < .001) sub-scales.

There were no group differences on either sub-scale before the video (positive *F*(2, 89) = 1.38, *p* > .1; negative *F*(2, 89) = .40, *p* > .1). However, group differences were apparent after the video (positive sub-scale *F*(2, 89) = 11.00, *p* < .001; negative sub-scale *F*(2, 89) = 21. 94, *p* < .001). Post-hoc LSD (Least Significant Difference) contrasts confirmed that, after watching the video, PANAS positive sub-scale ratings were higher in both the alcohol-positive and alcohol-negative groups compared to the neutral group (*p* < .01), although alcohol-positive and alcohol-negative groups did not differ from each other (*p* = .1). LSD contrasts on negative sub-scale ratings after the video revealed higher ratings in the alcohol-negative group compared to the alcohol-positive and neutral groups (*ps* < .01), although the latter groups did not differ from each other (*p* > .1).

We also performed within-subject t-tests, separately on each group, in order to identify changes in positive and negative affect that occurred as a result of watching the videos. In the alcohol-positive group, positive affect did not change (*t*(29) = 1.82, *p* > .08), but negative affect decreased *(t*(29) = 2.63, *p* < . 05) after watching the video. In the alcohol-negative group, positive affect did not change (*t*(29) = 1.17, *p* > .1), whereas negative affect increased (*t*(29) = 4.72, *p* < .001) after watching the video. In the control group, both positive affect (*t*(29) = 4.96, *p* < .01) and negative affect (*t*(29) = 4.05, *p* < .01) decreased after watching the video.

Effects of video manipulation on video questionnaire (Table S2b)

Participants’ responses were generally as anticipated – the alcohol-negative video was perceived as depicting the negative aspects of drinking, the alcohol-positive video was perceived as depicting the positive aspects of drinking, and the neutral video was perceived as boring and uninteresting. Each item was analysed using a separate one-way ANOVA, and each of these revealed a highly significant main effect of group (*Fs* > 55.65, *ps* < .001). Significant differences between conditions (LSD contrasts) are highlighted in the table.

Correlations with individual differences

We used non-parametric Spearman correlations to investigate the relationships between weekly alcohol consumption, AUDIT scores, scores on the three sub-scales of the AAAQ after watching the video, and ‘approach alcohol’ and ‘avoid alcohol’ indices (difference scores) derived from the SRC task. Both weekly alcohol consumption and scores on the AUDIT were positively correlated with scores on both the inclined-indulgent and obsessed-compelled sub-scales of the AAAQ (*rs* > .29, *ps* < .01), but were unrelated to scores on the resolved-regulated sub-scale (*ps* > .1), or to approach alcohol or avoid alcohol indices from the SRC task (*ps* > .09). The AAAQ sub-scales were unrelated to performance on the SRC task (*ps* > .1).

Study 3

Effects of video manipulation on PANAS ratings (Table S1c)

PANAS ratings were analysed using a 2 x 2 x 2 x 2 mixed design ANOVA, with within-subject factors of sub-scale (2: positive, negative), time (2: before video, after video), and between-subject factors of video group (2: alcohol-positive, alcohol-negative), and thought suppression group (2: thought suppression, control). The three way interaction sub-scale x time x video group was statistically significant (*F*(1, 96) = 13.92, *p* < .001), but the four way interaction sub-scale x time x video group x thought suppression group was not (*F*(1, 96) = 0.00, *p*  = 1). The three way interaction remained significant after adding AUDIT scores and weekly alcohol consumption as covariates (*F*(1, 94) = 11.05, *p* < .001). Subsequent post-hoc ANOVAs confirmed that the time x video group interaction was significant for both the positive (*F*(1, 98) = 4.79, *p* < .05) and negative (*F*(1, 98) = 14.50, *p* < .001) sub-scales.

There were no group differences on either sub-scale before the video (positive sub-scale *t(*98) = .56, *p* > .1; negative sub-scale *t*(98) = 1.08, *p* > .1). However, group differences were apparent after the video (positive sub-scale *t*(98) = 1.80, *p* < .05 negative sub-scale *t*(98) = 3.23, *p* < .001). As expected, scores on the positive sub-scale after watching the video were higher in the alcohol positive video group compared to the alcohol negative video group, whereas these group differences were reversed for scores on the negative sub-scale. Within-subject t-tests performed on data from the alcohol-positive video group revealed no significant change in either positive or negative PANAS sub-scale ratings after watching the video (*t*(49) = 1.05, *p* > .1 and *t*(49) = 1.44, *p* = .08). Whereas in the alcohol-negative video group, scores on the positive PANAS sub-scale decreased (*t*(49) = 2.71, *p* < .01) and scores on the negative PANAS sub-scale increased (*t*(49) = 3.59, *p* < .001), after watching the video.

Effects of video manipulation on video questionnaire (Table S2c)

Each item was analysed using a separate independent groups t-test, all of which revealed significant group differences (*ts* > 1.67, *ps* < .05). Responses were generally as anticipated – the alcohol negative video was perceived as depicting the negative aspects of drinking, the alcohol positive video was perceived as depicting the positive aspects of drinking, The positive video was also rated as less interesting and more boring than the negative video.

Correlations with individual differences

We used non-parametric Spearman correlations to investigate the relationships between weekly alcohol consumption, AUDIT scores, scores on the three sub-scales of the AAAQ after watching the video, and ‘approach alcohol’ and ‘avoid alcohol’ indices (difference scores) derived from the SRC task. Weekly alcohol consumption was positively correlated with scores on both the inclined-indulgent and obsessed-compelled sub-scales of the AAAQ (*rs* > .30, *ps*  < .01), but was not correlated with scores on the resolved-regulated sub-scale (*ps* > .1), or to approach alcohol or avoid alcohol indices from the SRC task (*ps* > .1). AUDIT scores were unrelated to any of the AAAQ sub-scales (*ps* > .1), and the AAAQ sub-scales were unrelated to performance on the SRC task (*ps* > .1).

Table S1a: PANAS ratings from study 1. Values are means ± SD

Alcohol pos Alcohol neg Neutral

PANAS positive (pre) 31.23 ± 8.21 30.30 ± 8.43 28.10 ± 5.54

PANAS positive (post) 33.07 ± 9.61 29.38 ± 8.68 22.87 ± 7.09

PANAS negative (pre) 13.47 ± 4.73 14.37 ± 4.57 13.53 ± 3.57

PANAS negative (post) 11.83 ± 2.81 18.03 ± 6.49 11.50 ± 2.36

Table S1b: PANAS ratings from study 2. Values are means ± SD

Alcohol pos Alcohol neg Neutral

PANAS positive (pre) 28.69 ± 6.04 26.66 ± 6.83 29.17 ± 7.13

PANAS positive (post) 28.07 ± 7.28 25.28 ± 7.14 22.52 ± 5.75

PANAS negative (pre) 12.31 ± 3.11 12.53 ± 4.03 11.83 ± 2.47

PANAS negative (post) 11.72 ± 3.61 15.59 ± 5.24 10.90 ± 1.37

Table S1c: PANAS ratings from study 3. Values are means ± SD

Alcohol pos Alcohol neg

PANAS positive (pre) 29.90 ± 6.82 29.02 ± 8.72

PANAS positive (post) 31.00 ± 10.63 27.52 ± 8.62

PANAS negative (pre) 13.02 ± 4.00 14.24 ± 6.96

PANAS negative (post) 12.48 ± 3.98 16.42 ± 7.51

Table S2a: Responses on the video questionnaire in study 1. Values range between 1 (strongly disagree) and 5 (strongly agree). Values are means (± SD) Alcohol positive Alcohol negative Neutral

‘This video could be used as used as an effective alcohol prevention video’^1^ 1.38 (.68) 4.16 (.81) 1.48 (.91)

‘I found this video interesting’^4^ 3.59 (.78) 4.00 (.80) 1.45 (.74)

‘This video adequately represented the negative aspects of alcohol consumption’ ^1^ 1.28 (.53) 4.10 (1.05) 1.21 (.62)

‘This video made me think about the positive aspects of alcohol consumption’ ^2^ 4.17 (.81) 1.63 (1.07) 1.31 (.60)

‘This video adequately represented the positive aspects of alcohol consumption’ ^2^ 3.97 (.78) 1.38 (.94) 1.17 (.60)

‘In my opinion, this video was wearisome / boring’ ^3^ 2.14 (.95) 1.94 (.56) 4.38 (.68)

‘This video could be used as an effective alcohol advertisement’ ^4^ 4.00 (.85) 1.81 (1.23) 1.21 (.49)

‘This video made me think about the negative aspects of alcohol consumption’ ^4^ 1.69 (.97) 4.44 (1.11) 1.17 (.54)

Key: ^1^ = alcohol-negative group differs from both alcohol-positive and neutral groups. ^2^ alcohol-positive group differs from both alcohol-negative and neutral groups. ^3^ neutral differs from both alcohol-positive and alcohol-negative groups ^4^ all groups differ from each other

Table S2b: Responses on the video questionnaire in study 2. Values range between 1 (strongly disagree) and 5 (strongly agree). Values are means (± SD)

Alcohol positive Alcohol negative Neutral

‘This video could be used as used as an effective alcohol prevention video’^1^ 1.43 (.73) 4.23 (1.01) 1.60 (.93)

‘I found this video interesting’^4^ 3.73 (.83) 4.20 (.61) 1.73 (.91)

‘This video adequately represented the negative aspects of alcohol consumption’ ^1^ 1.40 (.50) 4.27 (.83) 1.17 (.46)

‘This video made me think about the positive aspects of alcohol consumption’ ^4^ 4.30 (.70) 2.17 (1.39) 1.37 (.67)

‘This video adequately represented the positive aspects of alcohol consumption’ ^2^ 4.00 (.70) 1.47 (.82) 1.30 (.60)

‘In my opinion, this video was wearisome / boring’ ^3^ 2.17 (.95) 1.70 (.70) 4.13 (1.14)

‘This video could be used as an effective alcohol advertisement’ ^4^ 4.10 (.92) 2.33 (1.40) 1.33 (.61)

‘This video made me think about the negative aspects of alcohol consumption’ ^1^ 1.60 (.72) 4.43 (.90) 1.40 (.72)

Key: ^1^ = alcohol-negative group differs from both alcohol-positive and neutral groups. ^2^ alcohol-positive group differs from both alcohol-negative and neutral groups. ^3^ neutral differs from both alcohol-positive and alcohol-negative groups ^4^ all groups differ from each other

Table S3c: Responses on the video questionnaire in study 3. Values range between 1 (strongly disagree) and 5 (strongly agree). Values are means (± SD)

Alcohol positive Alcohol negative

‘This video could be used as used as an effective alcohol prevention video’ 1.48 (.76) 4.20 (1.01)

‘I found this video interesting’ 3.48 (.93) 3.88 (.87)

‘This video adequately represented the negative aspects of alcohol consumption’ 1.52 (.65) 4.22 (.84)

‘This video made me think about the positive aspects of alcohol consumption’ 3.86 (.93) 1.64 (.78)

‘This video adequately represented the positive aspects of alcohol consumption’ 3.90 (.84) 1.42 (.61)

‘In my opinion, this video was wearisome / boring’ 2.28 (1.03) 1.96 (.88)

‘This video could be used as an effective alcohol advertisement’ 4.10 (.74) 1.78 (1.09)

‘This video made me think about the negative aspects of alcohol consumption’ 1.82 (.83) 4.34 (.72)

Note: alcohol positive and alcohol negative groups significantly different on all items (ts > 1.67, ps < .0.05).

Table S4: Results of combined analysis of AAAQ.

A. Change in subscales over time. Values are mean ± SD

Alcohol-positive Alcohol-negative

Before video After video *p* Before video After video *p*

Inclined-Indulgent (I-I) 4.07 ± 1.93 4.44 ± 2.13 < .001 3.73 ± 1.77 2.52 ± 1.73 < .001

Obsessed-Compelled (O-C) 0.85 ± 1.16 1.28 ± 1.57 < .001 0.80 ± 0.96 0.58 ± 0.83 < .001

Resolved-Regulated (R-R) 1.25 ± 1.27 0.99 ± 1.21 = .001 1.42 ± 1.28 2.45 ± 1.49 < .001

B. Intercorrelations between change scores (after video minus before video), separately for each group. Values are Pearson correlation co-efficients, two-tailed. Statistically significant correlations are in **bold**.

Alcohol-positive Alcohol-negative

I-I O-C R-R I-I O-C R-R

I-I - ***r* = .39, *p* < .001** ***r* = -.20, *p* < .05** I-I - *r* = .04, *p* = .72 ***r* = -.36, *p* < .001**

O-C - - ***r* = -.20, *p* < .05** O-C - - *r* = -.09, *p* = .35

Table S5: Results from hierarchical linear regression models that test the influence of video condition on change in AAAQ inclined-indulgent and obsessed-compelled subscales after statistically controlling for change in AAAQ resolved-regulated subscales, and vice versa.

| Model | DV | Predictors | *B* | *S.E.B* | *β* | R^2^ ∆ | p |
| --- | --- | --- | --- | --- | --- | --- | --- |
| 1 | Inclined-Indulgent (change score) | *Step 1*  Resolved-Regulated (change score)  *Step 2*  Video condition | -.50  -.46 | .06  .09 | -.47  -.28 | .22  .08 | <. 01  < .01 |
| 2 | Obsessed-compelled (change score) | *Step 1*  Resolved-Regulated (change score)  *Step 2*  Video condition | -.18  -.26 | .04  .06 | -.27  -.24 | .07  .06 | < .01  < .01 |
| 3 | Resolved-Regulated (change score) | *Step 1*  Inclined-Indulgent (change score)  *Step 2*  Video condition | -.44  -.09 | .05  .09 | -.47  -.06 | .22  < .01 | < .01  .30 |
| 4 | Resolved-Regulated (change score) | *Step 1*  Obsessed-compelled (change score)  *Step 2*  Video condition | -.40  .04 | .08  .09 | -.27  .03 | .07  < .01 | < .01  .66 |
